# Feasibility and Efficacy of Fusion Imaging Systems for Immediate Post Ablation Assessment of Liver Neoplasms: Protocol for a Rapid Systematic Review

**DOI:** 10.29337/ijsp.162

**Published:** 2021-09-17

**Authors:** Pragati Rai, Sarada Dakua, Julien Abinahed, Shidin Balakrishnan

**Affiliations:** 1 Department of Surgery, Hamad Medical Corporation, Doha, Qatar

**Keywords:** Liver neoplasms, Ablation techniques, Treatment outcomes, Ablative margin, Image fusion

## Abstract

**Introduction:**

Percutaneous thermal ablation is widely adopted as a curative treatment approach for unresectable liver neoplasms. Accurate immediate assessment of therapeutic response post-ablation is critical to achieve favourable outcomes. The conventional technique of side-by-side comparison of pre- and post-ablation scans is challenging and hence there is a need for improved methods, which will accurately evaluate the immediate post-therapeutic response.

**Objectives and Significance:**

This review summarizes the findings of studies investigating the feasibility and efficacy of the fusion imaging systems in the immediate post-operative assessment of the therapeutic response to thermal ablation in liver neoplasms. The findings could potentially empower the clinicians with updated knowledge of the state-of-the-art in the assessment of treatment response for unresectable liver neoplasms.

**Methods and Analysis:**

A rapid review will be performed on publicly available major electronic databases to identify articles reporting the feasibility and efficacy of the fusion imaging systems in the immediate assessment of the therapeutic response to thermal ablation in liver neoplasms. The risk of bias and quality of articles will be assessed using the Cochrane risk of bias tool 2.0 and Newcastle Ottawa tool.

**Ethics and Dissemination:**

Being a review, we do not anticipate the need for any approval from the Institutional Review Board. The outcomes of this study will be published in a peer-reviewed journal.

**Highlights:**

Evaluation of the therapeutic response in liver neoplasms immediately post-ablation is critical to achieve favourable patient outcomes. We will examine the feasibility and technical efficacy of different fusion imaging systems in assessing the immediate treatment response post-ablation. The findings are expected to guide the clinicians with updated knowledge on the state-of-the-art when assessing the immediate treatment response for unresectable liver neoplasms.

## 1. Introduction

Image-guided percutaneous ablation is being recognized as the curative option for unresectable liver neoplasms [1]. Patients treated with thermal ablation are usually associated with fewer postoperative complications, infection and reduced hospital stays as compared to those undergoing surgical resection [2]. Despite potential advantages, the therapeutic effect of ablation is hampered by high rates of local tumor progression (LTP) [3,4,5,6]. Factors leading to LTP include suboptimal ablative safety margin, large tumor size, tumor proximity to the blood vessel, subcapsular location and adhesion of viable tumor cells to the RFA electrodes [7].

Since intraoperative histological confirmation of the ablation zone is not possible, it becomes critical to evaluate the therapeutic outcome of the ablation immediately post-ablation to achieve the most favourable outcomes [8]. Conventionally, the therapeutic outcome of ablation is evaluated through the presence/absence of residual tumor on first follow-up or the absence of LTP in subsequent follow-up imaging [9]. Hence, an immediate intraprocedural technique to predict the treatment success would be highly desirable, due its potential clinical impact [8]. This would allow the clinicians to immediately assess the treatment response post-procedure and perform immediate supplementary ablation if needed. This potentially avoids the need for secondary treatment as well as reducing the economic burden to the patients [10].

A crucial step in the curative treatment of liver neoplasms is the evaluation of treatment success [11]. Pre-ablation planning using volumetric assessment from Computed tomography(CT)/Magnetic resonance(MR) images can positively impact treatment success [12,13,14]. However, immediate post-ablative assessment would provide a more accurate clinical picture on the efficacy of the treatment. Accumulating evidence suggests that ablative margin (AM) is an independent predictor of LTP and a critical determinant affecting treatment success [15,16,17]. There have been efforts to assess AM by manual and visual comparison of pre- and post-ablation two-dimensional (2D) images [18,19]. However, this technique is subjective and prone to errors and can be challenging even for experienced radiologists. Hence, there is a clear need for methods for the precise evaluation of the AM immediately after ablation [20].

In recent years, novel imaging methods have emerged, which utilize image fusion techniques for post-ablation assessment, which can then be visualized using augmented- and mixed- reality devices [21]. These fusion techniques include CT-CT, MR-MR, ultrasound (US)-CT/MR and contrast-enhanced ultrasound (CEUS)-US in the assessment of AM [22,23,24]. New emerging supportive software platforms are also available that reliably and quickly assess the ablation areas and the extent of the AM. In these emerging platforms, the pre- and post-ablation images are merged using either rigid or nonrigid registration software [25,26]. Image fusion using commercially available registration software also assess the AM that can be used as an intraoperative tool to evaluate the treatment efficacy of ablation procedures [27]. This work aims to summarise studies investigating the feasibility and efficacy of the fusion imaging systems for the immediate assessment of the therapeutic response to thermal ablation in liver neoplasms.

## 2. Objectives and significance

Tumor-free margin as well as the complete eradication of microscopic invasion around the pathological periphery is critical for an optimal treatment outcome while maintaining liver function. Therefore, immediate accurate assessment of the ablative safety margin is a crucial step in clinical practice, as it may influence the following treatment or follow-up strategy. We propose a rapid review overviewing the feasibility and efficacy of the fusion imaging systems in the immediate assessment of the therapeutic response to thermal ablation in liver neoplasms. It is expected that the findings of this study could potentially empower the clinicians with updated knowledge of the state-of-the-art image fusion techniques in the assessment of treatment response for unresectable liver neoplasms immediately post ablation.

In this regard, we shall attempt to respond to the following questions:

- Are fusion imaging systems feasible for assessing the immediate therapeutic response to thermal ablation in liver neoplasms?
- Do fusion imaging systems accurately detect the AM to determine the technical efficacy of thermal ablation in liver neoplasms?

## 3. Methods and analysis

### 3.1 Study design

This review will be designed and implemented based on the Preferred Reporting Items for Systematic Reviews and Meta-Analyses (PRISMA) guidelines [28]. The research question is formulated within the PICO tool, as follows:

- (P) Participants: Liver neoplasms (primary or secondary) treated with Thermal ablation [Radiofrequency ablation (RFA)/Microwave ablation (MWA)].
- (I) Intervention: Assessment of therapeutic response in liver neoplasms immediately post ablation by fusion of pre-(CT or MR or US) ablation and post (CT or MRI or CEUS) ablation images. Qualitative and quantitative assessment of AM done immediately at the end of ablation by fusion imaging systems.
- (C) Comparator: None.
- (O) Outcome: Feasibility and technical efficacy of fusion imaging systems for assessment of AM immediately after percutaneous thermal ablation of liver neoplasms.

This rapid review protocol has been registered in the International Prospective Register for Systematic Reviews and Meta-analysis (PROSPERO) with the registration number CRD42021265980.

### 3.2 Eligibility criteria

For this review, we will consider studies following experimental as well as observational study design, specifically controlled trials (randomized and non-randomized), cohort studies (prospective and retrospective), and analytical case-control and cross-sectional studies. Based on preliminary analysis, the authors determined the time for the search to be from January 2016 to June 2021. From these, those studies will be included which specifically assess the immediate therapeutic response to percutaneous thermal ablation in liver neoplasms using fusion imaging systems. Any descriptive or non-analytical observational studies/commentaries will be discarded, including case reports/series, review articles, letters, consensus statements and opinions. Additionally, liver neoplasms treated with techniques other than RFA and MWA will be excluded. Articles published in languages other than English will be not be included.

### 3.3 Search strategy

The electronic database MEDLINE (via PUBMED), EMBASE and Cochrane Library Central Registry databases will be extensively searched to retrieve articles related to the application of fusion imaging in the evaluation of immediate therapeutic response to ablation for liver neoplasms. Full-text papers will be obtained after the abstracts are evaluated for relevance. The reference lists of included articles will be manually searched to identify any additional studies. To maximize the sensitivity of the search, we will use both Medical Subject Headings (MeSH) and keyword searches. The search terms “liver neoplasms’’ [MeSH Terms], “carcinoma, hepatocellular’’ [MeSH Terms], “ablation techniques” [MeSH Terms], “treatment outcome” [MeSH Terms] ‘ablative margin’, ‘fusion imaging’, ‘intraprocedural’, ‘three dimensional’ and ‘volumetric assessment’ will be used in combination with the Boolean operators AND or OR.

### 3.4. Study selection

After applying the search criteria above, selected articles will be exported to Covidence software and any duplicates will be removed [29]. Three reviewers (PR, SB, SD) will independently screen title and abstract of the studies based on the predefined inclusion and exclusion criteria. Regular meetings will be held to discuss potential conflicts, and any disagreements will be resolved through discussion with a fourth reviewer (JA).

Following that, full text of selected papers will be screened for further selection. Reasons for exclusion of full-text papers that do not fulfil the inclusion criteria will be recorded and reported in the review. The final selected list of articles will concurrently be evaluated by the fourth reviewer (JA) against the predefined inclusion criteria, and any conflicts would be resolved through discussions. The results of the search will be reported in the final review and presented as a PRISMA flow diagram.

### 3.5. Data extraction

Data from selected articles will be assessed and analyzed independently by three reviewers (PR, SB, SD) using the data extraction capabilities of the Covidence platform. Data extraction will be based on a set of predefined variables/labels. From each selected study, baseline patient characteristics (age, tumor type, tumor size), study design, ablation type, type of fusion modality employed, technical efficacy, time taken for fusion of pre- and post-ablation images, software used for assessment of AM), complete ablation rate, supplementary ablation rate, follow up time, imaging modality used during follow-up and LTP rates will be recorded. Any modifications to the extraction protocol will be discussed with the fourth reviewer (JA).

### 3.6. Assessment of risk of bias

The observational studies will be critically evaluated for methodological quality using the Newcastle Ottawa scale [30]. The Cochrane risk of bias 2.0 tool will be used to assess the risk of bias for interventional studies [31]. If required, the authors of the papers would be contacted for any additional clarifications or data. The results of critical appraisal will be reported in risk of bias summary and table. Any disagreements or discordance amongst the authors will be resolved through discussion with the fourth author (JA).

### 3.7. Data presentation and data synthesis

The extracted data will be presented in diagrammatic and tabular format in a manner that aligns with the objective of this review. A narrative synthesis of the findings will be provided from the included studies, structured around the feasibility and technical efficacy of different fusion imaging systems for the immediate assessment of AM post-ablation in liver neoplasms. Differences in outcomes based on patient characteristics (age, tumor type, tumor size) would be highlighted. Comparative synthesis of ablation type and type of fusion modality employed would be undertaken, and their impact on technical efficacy and time taken would be analyzed. Any influence of software used for assessment of AM on complete ablation rate, supplementary ablation rate would be analyzed. Duration of follow-up, imaging modality used during follow-up and LTP rates will be explored.

## 4. Ethics and dissemination

This review uses peer reviewed published literature and attempts to summarize their findings narratively; as a result, we do not anticipate any need for an institutional review board approval. The results of this study will be summarised in English and will be published in a peer-reviewed scientific publication and disseminated on research platforms in accordance with copyright regulations.

## 5. Discussion

### 5.1. Current implications of the study

Tissue changes at the ablative site evolves with time after the procedure. This makes it challenging to accurately assess the post-ablative efficacy of treatment as more time passes after the procedure. Tumor-free margin and total eradication of microscopic invasion around the pathological periphery is critical for optimal treatment outcome while maintaining hepatic function. Therefore, immediate accurate assessment of the ablative safety margin is crucial in clinical practice, as it may impact the subsequent treatment or follow-up strategy

The findings of the current review will potentially empower the clinicians with updated knowledge of the state-of-the-art image fusion techniques in the assessment of treatment response for unresectable liver neoplasms immediately post-ablation. Application of fusion of pre- and post-interventional scans in conjunction with an assessment of the AM immediately post-ablation can possibly prevent any errors and objectively evaluate the technical success of the procedure. This approach could have a potential clinical impact in identifying possible sub-total ablation and the need for re-treatment within the same operative session. By reducing additional treatment sessions, it could improve patient’s quality of life, decrease stress, and ease their financial burden as well as optimize usage of hospital resources.

### 5.2. Limitations of the study

The study is anticipated to have some limitations in terms of concurrent clinical practice and research. There is limited analytic literature reported on the immediate post-operative assessment of AM after ablation using fusion imaging techniques, which would directly affect our synthesis. The different fusion imaging techniques used to assess immediate post-ablative margins have yet to reach consensus and standardization among clinicians. Only a few comparative studies between the different fusion imaging techniques have been done to assess immediate post therapeutic response. There is also a lack of consensus on a standardized protocol between clinicians for the same registration software used for the quantitative assessment of AM. The current protocol did not incorporate grey literature, where the most current and updated emerging techniques are first published. Further research is also required to validate the use of existing commercial co-registration software (Ablation-fit TM [32], Hepacare [33] etc.) to assess AM intra-procedurally for a broader group of tumour types (subcapsular tumors, subphrenic tumors, etc.) and ablation procedures (cryoablation, laser ablation, etc).

Finally, despite technological advancements, fusion registration still faces technical challenges and limitations with the existing methods in the setting of organ motion induced by breathing or positional changes [34,35,36,37,38,39]. These challenges include (a) different physical acquisition processes, which may generate a statistical correlation between imaging structures that do not correspond to the same anatomical structures, violating one of the underlying assumptions for most intensity-based similarity measures, and (b) the deformation, spatial and temporal variabilities.

### 5.3. Future Implications of the study

The study could potentially highlight areas of future research addressing primarily the technical limitations due to deformation, spatial and temporal variabilities, to gain widespread clinical acceptance of robust techniques. The ability to capture complex image deformations and establish accurate pointwise correspondence is key to many clinical applications of computer vision that involve image fusion and atlas construction. These properties become particularly challenging when the object depicted on the images undergoes a severe deformation or has in general a high shape variability. Addressing dissimilarities due to inter- and intra-fractional anatomical variations from the pre-operative image set can further augment accuracy of the technical fusion pipeline, empowering widespread use in clinical practice. Researching techniques to homogenize multi-modality, multi-temporality, domain specificity and parameter sensitivity may facilitate image processing algorithms for segmentation, rigid and non-rigid co-registration, and volume analysis devoted to intraprocedural quantitative assessment of AM. This study could also highlight research focus for the refinement of breathing synchronization devices and automatic recognition and registration of hepatic vessels.

## 6. Amendments

Any amendments to this protocol will be prospectively updated on the PROSPERO International Prospective Register of Systematic Reviews.

## Abbreviations

PRISMA-P: Preferred Reporting Items for Systematic Review and Meta-Analysis Protocols

AM: Ablative margin

LTP: Local tumor progression

CT: Computed tomography

MR: Magnetic resonance

US: Ultrasound

CEUS: Contrast-enhanced ultrasound
